# Optical Detection of Cancer Cells Using Lab-on-a-Chip

**DOI:** 10.3390/bios13040439

**Published:** 2023-03-30

**Authors:** Luis Abraham García-Hernández, Eduardo Martínez-Martínez, Denni Pazos-Solís, Javier Aguado-Preciado, Ateet Dutt, Abraham Ulises Chávez-Ramírez, Brian Korgel, Ashutosh Sharma, Goldie Oza

**Affiliations:** 1 Centro de Investigación y Desarrollo Tecnológico en Electroquímica, Parque Tecnológico Querétaro, Pedro Escobedo, Querétaro C.P. 76703, Mexico; 2 Instituto Nacional de Medicina Genómica, Mexico City C.P. 14610, Mexico; 3 Tecnologico de Monterrey, School of Engineering and Sciences, Centre of Bioengineering, Campus Queretaro, Querétaro C.P. 76130, Mexico; 4 Instituto de Investigaciones en Materiales, Circuito Exterior S/N Ciudad Universitaria, Mexico City C.P. 04510, Mexico; 5 McKetta Department of Chemical Engineering and Texas Materials Institute, The University of Texas at Austin, Austin, TX 78712-1062, USA

**Keywords:** lab-on-a-chip, optical detection, cancer, fluorescence, chemiluminescence, surface plasmon resonance SPR, surface-enhanced Raman scattering SERS

## Abstract

The global need for accurate and efficient cancer cell detection in biomedicine and clinical diagnosis has driven extensive research and technological development in the field. Precision, high-throughput, non-invasive separation, detection, and classification of individual cells are critical requirements for successful technology. Lab-on-a-chip devices offer enormous potential for solving biological and medical problems and have become a priority research area for microanalysis and manipulating cells. This paper reviews recent developments in the detection of cancer cells using the microfluidics-based lab-on-a-chip method, focusing on describing and explaining techniques that use optical phenomena and a plethora of probes for sensing, amplification, and immobilization. The paper describes how optics are applied in each experimental method, highlighting their advantages and disadvantages. The discussion includes a summary of current challenges and prospects for cancer diagnosis.

## 1. Introduction

Cancer is a pressing issue in modern medicine, with millions of people worldwide affected by this disease. According to GLOBOCAN, approximately 2.2 million cases of infection-attributable cancer have been diagnosed globally in 2018, encompassing several types of cancer [1]. Given its global significance, accurate diagnosis is crucial in the fight against cancer. This requires the development of new methods that can differentiate between the different types of cancer and provide personalized treatments for each patient [2]. To address this, a promising approach is using microfluidic techniques incorporated in lab-on-a-chip devices, which have been applied in biomedical research and medical treatment. These devices offer a favorable solution for efficiently and precisely detecting cancer cells [3,4].

The early detection and diagnosis of cancer cells are crucial in guiding effective treatment strategies [5]. Separating and sorting cancer cells allow healthcare professionals to expand their treatment options and gain insight into a patient’s condition. These efforts increase the accuracy of diagnoses and optimize therapeutic outcomes. Over the past decade, researchers have made considerable progress in identifying and isolating cancer-related biomarkers in various molecules and tissues, such as DNA, miRNA, proteins, and tumor cells, as potential indicators of cancer in the blood or tissues [6]. One study that exemplifies this progress is the work of Zhou et al., who used a microfluidic channel to isolate circulating tumor cells in patients with lung cancer [7].

Circulating tumor cells (CTCs) detection and isolation are critical in understanding and treating cancer. CTCs are rare cells found in a patient’s blood and provide information about the presence and progression of cancer before a primary tumor can be detected [8]. The study of CTCs has proven valuable in predicting metastasis, prognosis, and therapeutic response in various types of cancer [9]. Moreover, separating cancer cells from normal ones has become an essential aspect of lab-on-a-chip devices [10]. In recent years, microfluidic techniques, such as dielectrophoresis, magnetic, acoustic, and passive microfluidic methods have emerged as promising methods for identifying and separating cancer cells [11].

Microfluidic technology is a rapidly growing field that aims to study and manipulate fluids at a microscale. The concept is based on the precise control of fluid flow in micro and nano channels, allowing for accurate monitoring of fluidic behavior [12]. The behavior of fluids at the microscale can differ significantly from that of macroscale fluids, as observed in parameters, such as resistance to flow and surface tension [13]. This has driven numerous advancements in the field of microfluidics and its applications [14]. This technology involves the manipulation of fluids in micro- and nano-liter quantities through channels with dimensions ranging from 100 nm to 500 μm. This field of investigation and development offers unique opportunities for precise control over reaction and mixing rates due to the low Reynolds numbers in microfluidic systems and the resulting laminar flow behavior. Over the years, microfluidics has been applied in various domains, from automated screening to lab-on-a-chip and tissue-on-a-chip devices, leading to a revolution in molecular marker detection for disease monitoring [15].

Biosensors are analytical devices that convert biological responses into measurable signals, allowing the detection and quantification of analytes of interest with high sensitivity and specificity [16]. Among the various transduction methods, optical detection techniques have been widely utilized in biosensing due to their versatility, non-invasiveness, and potential for miniaturization. Optical biosensors exploit the interaction between light and biological molecules to generate a signal that can be quantified and related to the concentration of the target analyte [17].

This review paper aims to provide an overview of optical methods in combination with lab-on-a-chip techniques for detecting cancer cells. We will discuss each optical method’s physical description and highlight its advantages and disadvantages in conjunction with microfluidic devices.

## 2. Optical-Based Detection

**Figure 1:**
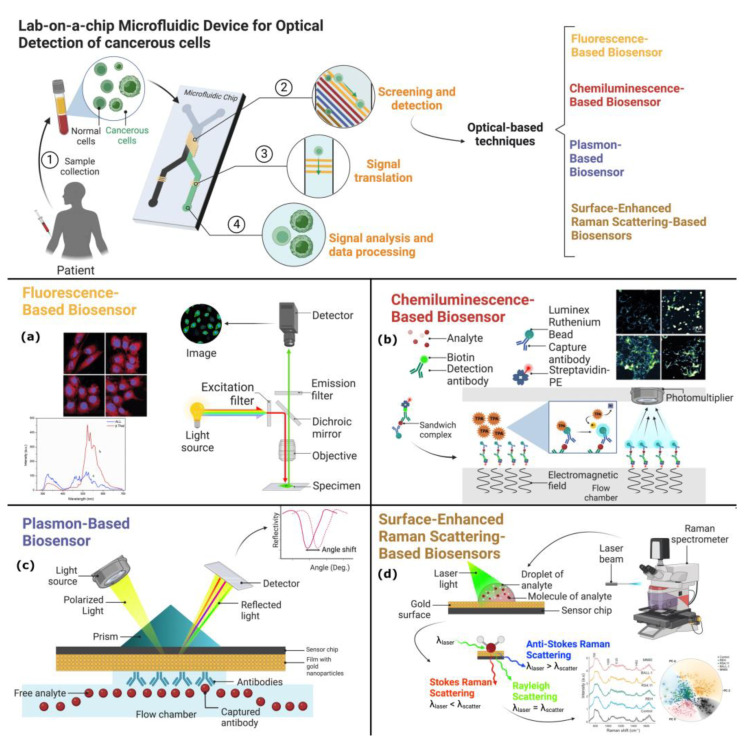
The schematic diagram displays lab-on-a-chip microfluidic devices utilizing optical-based detection techniques. At the top, a standard microfluidic channel is shown, where a human sample containing normal and cancerous cells has been introduced. The cells pass through microchannels, and a screening and detection step is performed using an optical-based detection technique. The four types of sensor chips depicted are (**a**) fluorescence-based biosensor, (**b**) chemiluminescence-based biosensor, (**c**) localized surface plasmon resonance, and (**d**) surface-enhanced Raman scattering sensor chips. Insets of micrographs adapted with permission from Refs. [20,21] a and b, respectively and [22] for d. Copyright 2019 WILEY and 2019 and 2021 Elsevier. (Created with Biorender.com).

Different types of optical-based biosensors, including fluorescence, Raman, refraction, phosphorescence, absorbance, scattering, and more, have been developed and applied to various biological and clinical applications [17,18]. Optical biosensors offer numerous advantages, such as high sensitivity, real-time detection, label-free analysis, low cost, and small form factor [19]. These features make them attractive for integration into lab-on-a-chip devices, which aim to perform sample preparation, research, and detection in a miniaturized and automated format. In Figure 1, the four primary optical methods discussed in this paper are presented, along with their most prominent features. In the following, we will review the latest developments for these optical-based biosensors and their incorporation into lab-on-a-chip devices, emphasizing their potential uses in the identification and clinical diagnosis of cancer as well as point-of-care (POC) testing.

## 3. Fluorescence-Based Biosensors

Fluorescence-based biosensors are a powerful tool for detecting biomolecules, as they can be utilized to measure the concentration, localization, and dynamic changes of such species. This is achieved through the phenomenon of fluorescence, in which electromagnetic radiation is absorbed by fluorescent molecules, resulting in the emission of fluorescent light [19]. Fluorescent molecules that label biomolecules include dyes, fluorescent proteins, and quantum dots. The main approaches of fluorescence biosensors include fluorescent turn-off/on, fluorescent enhancement, and fluorescent resonance energy transfer (FRET). To enable these measurements, fluorescence-based biosensors utilize excitation light sources, such as LEDs or lasers, and photodetectors capable of recording changes in fluorescence intensity. As a result of their high sensitivity and rapid response time, fluorescence-based biosensors have found widespread application in medical diagnostics and the monitoring of the quality of food and the environment.

**Figure 2:**
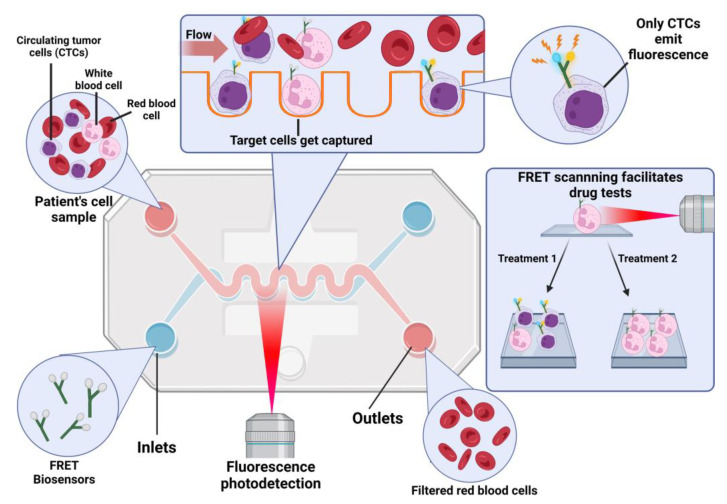
A lab-on-a-chip fluorescence device for biosensing. The device consists of microchannels and sensing regions that allow for the detection and analysis of specific biomolecules in biological samples. Fluorescent probes are used to detect the presence of these biomolecules, and the fluorescence signal is read by a detector integrated into the chip. Created with Biorender.com.

FRET-based optical sensors can detect changes at the scale of angstroms to nanometers [23]. However, conventional fluorescent molecules, such as organic dyes, present limitations as they can be toxic and undergo photobleaching. Incorporating nanomaterials into the development of fluorescent devices has provided a solution to these challenges, enabling the creation of low-cost, portable sensors with enhanced fluorescence signals by fluorescence signal-enhancing nanostructures. Using FRET assays and fluorescence lifetime flow cytometry, it was possible to detect phosphorylated EGFR in tumor cells [24]. These results are a step forward in the development of devices capable of measuring subcellular phenomena relevant to cancer research. In Figure 2, a schematic of a microfluidics device for biosensing using the FRET technique is presented.

Accurately identifying the phenotypic characteristics of primary cancer through the examination of CTCs is crucial for personalized treatment and understanding the behavior of cancer. To overcome the limitations of isolating and analyzing CTCs, microfluidic platforms integrated with fluorescence detection systems have been widely adopted for multiplexed analysis. By directly capturing biomolecules and anchoring CTCs in a specific section of the microfluidic device, the use of fluorescent-dye labeled antibodies allows for the efficient washing and analysis of the captured CTCs. Among the most common applications of fluorescence-based detection is the microfabrication of devices that capture cells based on surface markers or the quantification of biomarkers in serum [25,26,27]. The simplest design includes the coating of microchannels of molecules with a certain affinity to CTCs or the use of antibodies directed to antigens of the cell surface. Once CTCs are captured, these devices can be used to test drug susceptibility for a personalized treatment [28]. These kinds of devices have also been used with cancer cell spheroids coupled to multicolor microscopy [29]. Taking advantage of microfluidics, it was possible to separate single cells and promote the formation of single-cell-derived colonies for drug testing. An array with more than 4000 microchambers coupled to a concentration gradient generator allowed researchers to distinguish cell heterogeneity of both a leukemia cell line and primary tumor cells [30]. The device enables researchers to monitor cell viability over several time points by registering the fluorescence of live and dead cells.

Chiu et al. developed a microfluidic device for the label-free detection of CTCs based on their production of lactic acid, avoiding bias from heterogeneous cancer cell surface antigens [31]. The device generates uniform water-in-oil cell-encapsulating microdroplets, enabling fluorescence-based optical detection of lactic acid production. The detected signals were proportional to the number of cancer cells within the microdroplets but were insensitive to leukocytes. Validation tests confirmed the accurate detection of cancer cells in cell suspensions. This study proposes a promising label-free method for detecting live CTCs in blood circulation. Other examples of this approach include the quantification of metabolic intrinsic fluorescent co-factors, such as nicotinamide adenine dinucleotide (NADH) and nicotinamide adenine dinucleotide phosphate (NADPH). This label-free method allowed researchers to distinguish the differential metabolic states of K-562 and Jurkat cell lines [32].

Heterogeneous cell behavior in cancer tumors is a characteristic that demands a methodological strategy that enables the analysis of cytosolic proteins at a single-cell level. To this end, microfluidics technology has been coupled with flow cytometry to better characterize the physical characteristics of CTCs, to understand metastasis processes, and to be able detect CTCs from blood samples [26,33]. For example, fluorescently labeled CTCs have been isolated from an unanesthetized mouse using a microfluidic sorter for RNA-Seq analysis [34]. The efficient cell sorting has been made possible through the development of different strategies including acoustic, magnetic, and piezoelectric mechanisms. The implementation of an on-chip piezoelectric actuator microvalve has proved to be useful for the enrichment of rare cells [35,36]. With this device, it was possible to enrich 0.6% of fluorescently labeled MCF-7 cells from a cell mixture to obtain a purity of 90% [35,36]. The development of an on-chip photoacoustic imaging flow cytometry has also shown promising results to detect and isolate CTCs in the blood. The integration of an optical excitation microfluidic chip and ultrasound detection in an on-chip device allowed researchers to apply multicolor illumination to differentiate melanoma cells in whole blood and in blood samples from mice [37]. The combination of fluorescence-activated cell sorting and deep learning has created the technology-denominated intelligent image-activated cell sorting. In preliminary testing, the machine was able to sort two different strains of budding yeast [38].

Imaging flow cytometry has been improved over the last decade and has provided new tools for the study of heterogenous cell populations. Among the main problems with imaging flow cytometry are the limited capacity of high-throughput, sensitivity, and spatial resolution [39,40]. Virtual-freezing fluorescence imaging combined with flow cytometry has been developed to increase the exposure time of the image sensor and provide a better signal-to-noise ratio as compared to other technologies [41]. The basic principle of the method is to digitally “freeze” an image of a flowing cell with the aid of a microfluidic chip, a speed-controlled polygon scanner, and a series of timing control circuits. This method scans over 10,000 cell per second, achieving images similar to fluorescence microscope and allows the classification of CTCs by antibody labelling (EpCAM^+^) and accumulation of protoporphyrin [41].

## 4. Chemiluminescence-Based Biosensors

Chemiluminescence is a process that releases light energy because of a chemical reaction. This phenomenon is activated by the oxidation of certain substances, such as reagents, intermediates, and fluorophores, which leads to a highly energetic oxidized intermediate [42]. When an intermediate undergoes a transfer or release of energy, it can cause nearby fluorophores to return to their ground state, resulting in luminescence [43]. There are two primary categories of chemiluminescence, direct and indirect, which utilize different chemical energy conversion mechanisms. The intensity of the resulting light emission can be measured using three different methods: (a) by statically mixing the reagents in front of the detector, (b) by immobilizing the chemiluminescent reagents on a solid support, such as filter paper, and allowing them to interact with the sample via diffusion or convection, or (c) by employing flow measurement systems [43,44].

Chemiluminescence measurement instruments consist of a mixing device and a detection system. Chemiluminescence-based biosensors are attractive due to their affordability, simplicity, low limit of detection, and a broad range of calibration [45]. However, they also have limitations, such as low sensitivity and limited selectivity, unless paired with a robust separation system. Chemiluminescence-based biosensors have various applications, including detecting biomarkers, toxins, metal ions, viruses, and bacteria [46]. The use of nanomaterials has expanded the range of applications and improved sensitivity [47].

Detecting tumor markers in human serum is crucial for early and effective diagnosis and treatment of certain tumors or carcinomas [48]. Immunoassay is commonly used for this purpose, but clinical analyses, such as FIA, ELISA, MS, and CLIA are often not feasible in developing countries due to their complexity and cost. Therefore, there is a need for alternative, simple, and low-cost detection methods. In their research, Wang et al. developed a sandwich-type electrogenerated chemiluminescence (ECL) immunosensor using a 3D microfluidic origami device for the determination of CA125 [49]. The device is low cost, portable, and disposable, and cyclic voltammetry was used for ECL detection. The study aimed to explore POC testing devices that are simple, sensitive, low-cost, and disposable.

The early diagnosis of thyroid cancer is crucial, and thyroid-stimulating hormone (TSH) is a widely used biomarker for this purpose [50]. However, conventional sandwich enzyme immunoassays (EIAs) with colorimetric detection cannot rapidly quantify TSH in human serum due to its low cut-off value. To improve sensitivity, fluorescence and chemiluminescence have been used as detection methods for EIAs. The 1,1’-oxalyldiimidazole chemiluminescence enzyme immunoassay (ODI-CLEIA) has been reported as a more sensitive method for quantifying cancer biomarkers [51]. Recently, a biosensor based on ODI-CLEIA was developed by Choi et al. that uses electrostatic interaction between protein and nanoparticle to detect trace levels of TSH in human serum [46]. The biosensor was found to be at least three times more rapid than a commercially available EIA. This biosensor could be used as a new method for the early diagnosis of thyroid cancer and could potentially lead to the development of a new lateral flow assay for public health. Additionally, the technology used to develop the biosensor could be applied to fabricate highly sensitive biosensors for the quantification of various cancer biomarkers and biomarkers for early diagnosis of human diseases.

**Figure 3:**
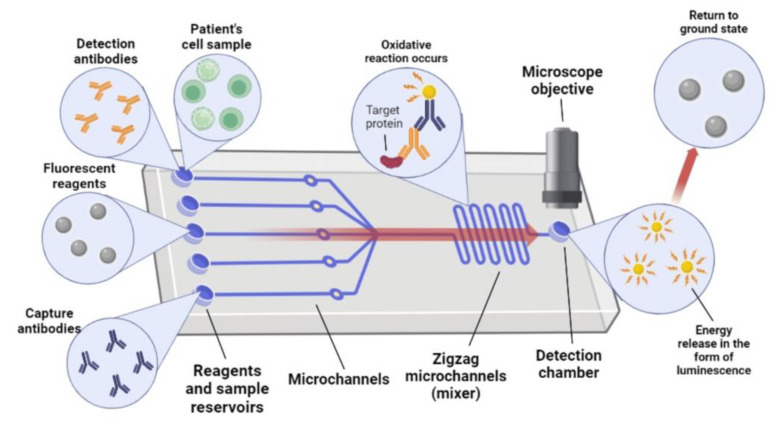
Schematic illustration of the microfluidic chemiluminescence biosensor. Created with Biorender.com.

Chemiluminescence has emerged as a promising technique for bioimaging and therapy due to its unique properties. However, several challenges still need to be addressed to develop synthesizable and activatable chemiluminescent platforms for effective photodynamic therapy. Additionally, chemiluminescence therapy systems activated by various disease-associated biomarkers and the exploration of chemiexcited photothermal therapy platforms are promising strategies for future oncotherapy. To address these challenges, Hu et al. have developed an automated diagnostic platform using microfluidics technology for POC testing [52]. The platform consists of a disposable microfluidic chip and a portable instrument that enables completely automated operations (see Figure 3). The chip contains patterned antibody/antigen stripes and was used to implement a chemiluminescence immunoassay for the quantitative detection of C-reactive protein and testosterone in real clinical samples. This lab-on-a-chip platform provides a promising strategy for POC diagnosis with features of quantitation, portability, and automation.

Microfluidic platforms have become an attractive technology for POC diagnostics due to their low cost, portability, and ability to perform highly sensitive assays [53]. However, the main challenges faced by these platforms are the precise transport of fluids in the microchannels and the development of automated instruments for sensitive detection of biochemical signals. Most platforms use peristaltic pumps to drive the fluid, but this can make the experimental operation more complicated. Centrifugal platforms have limitations as the radial path is limited by the radius of the centrifugal disks, and the chip cannot stop until the completion of the experiment. Microfluidic platforms using chemiluminescence methods for the quantitative detection of biomarkers have been developed, but they have limitations in terms of sensitivity and absolute quantification. In recent studies, researchers have developed novel systems that combine chemiluminescence detection with magnetic particles, resulting in improved sensitivity and enabling rapid assays in a microfluidic format [54]; see Table 1 for an overview.

In the study of Min et al., they report the development of an automated microfluidic chemiluminescence immunoassay platform for sensitive and quantitative detection of ferritin [59]. The platform employs a reliable and flexible suction method to drive liquid flow, effectively addressing the limitations of gas-driven systems and providing an external reference for those utilizing centrifugal flow drivers. The detection system can achieve quantitative analysis by detecting photon signals from acridine ester in alkaline conditions. The platform is both low-cost and easily produced through the use of laser cutting and hot press machines. Reagents are pre-loaded into the liquid storage chamber of the chip and are subsequently released through a flexible vacuum suction cup, powered by a pneumatic pump. Once the sample is introduced, the chip is inserted into a customized instrument, where chemiluminescence signals are acquired and processed. Within just 45 min, quantitative results can be obtained. Overall, this lab-on-a-chip platform boasts numerous advantages, including high sensitivity, quantitation capabilities, portability, and ease of use, making it a promising technology for use in diagnostic fields.

A compact and versatile analytical tool has been developed by Roda et al. for performing various types of bioassays simultaneously [60]. The microfluidics-based reaction chip coupled with a thermoelectrically cooled CCD sensor through a fiber optic taper enables high light collection efficiency and adequate spatial resolution within a portable device. The miniaturization of the reaction chamber ensures fast analysis times, while the use of chemiluminescence detection provides wide signal dynamic range and high detectability. The successful simultaneous quantification of an enzyme assay, a nucleic acid hybridization assay, and an immunoassay demonstrated that a range of analytes can be measured in a single run, enabling the realization of a complete, personalized diagnostic panel test for early disease diagnosis and patient follow-up.

In addition, Tang et al. developed a low-cost, 3D-printed microfluidic device for automated protein detection [61]. The device features three reagent reservoirs, a 3D network for passive mixing, and a transparent detection chamber that houses a glass antibody array for measuring chemiluminescence with a CCD camera. The prototype device was used for multiplexed detection of prostate cancer biomarkers, achieving detection limits of 0.5 pg/mL for PSA and PF-4 in diluted serum, with four orders of magnitude log dynamic ranges. The accuracy was validated by analyzing human serum samples, and this device has a good potential for further development as a POC cancer diagnostic tool.

## 5. Plasmon-Based Biosensors

Over the last 20 years, there has been a significant increase in technological advancement and research efforts directed toward developing optics-based surface plasmon resonance (SPR) sensors. These sensors determine the amount of the analyte by assessing the refractive index, absorbance, and fluorescence properties of the analyte molecules or the chemo-optic transduction medium [62]. This section will delve into the key aspects of lab-on-a-chip sensors that use SPR for biosensing.

This method has established itself as a critical tool for biosensing, with significant advancements in instrumentation and applications [63]. SPR has been successfully commercialized and is widely used for characterizing and quantifying biomolecular interactions. Here we will review the significant developments in SPR technology, highlight its key application in medical areas, and provide examples of its use in biosensing. SPR’s ability to measure biomolecular interactions in real-time, without the use of labels, makes it a valuable and powerful technique [64]. The changes in the refractive index near the metal-dielectric surface are detected by measuring the shift in the resonance angle [65].

Due to the resonating nature of surface plasmons, the local electromagnetic wave is magnified, leading to increased optical phenomena, such as absorption, scattering, transmission [66], and photoluminescence [67]. The localized field enhancement at the interface, which can extend hundreds of nanometers, is responsible for the significant optical effects observed [68]. Thanks to recent advancements in nanoscale material fabrication and manipulation, the surface plasmon modes generated by different metal nanostructures have been the subject of extensive research in recent years. [69].

Localized-surface-plasmon-resonance (LSPR)-based biosensors have gained popularity due to their label-free nature, low cost, multiplexing potential, portability, and ability to monitor various targets in real-time [70]. These biosensors rely on the collective oscillation of conduction electrons in metallic nanostructures, such as gold, silver, and copper, to generate absorbance bands that can be measured using UV-visible spectroscopy [71]. The LSPRs of gold and silver nanoparticles typically occur in the near-visible IR range and depend on various factors, such as the nanoparticles’ size, shape, and composition, as well as the orientation of the electric field relative to the nanoparticles and their dielectric properties in the surrounding medium [72]. Despite the promising features, fabricating these biosensors presents significant challenges, including minimizing non-specific binding, cross-reactivity of the target set, noise reduction, synthesizing stable nanoparticles, and selecting well-characterized bioreceptors.

**Figure 4:**
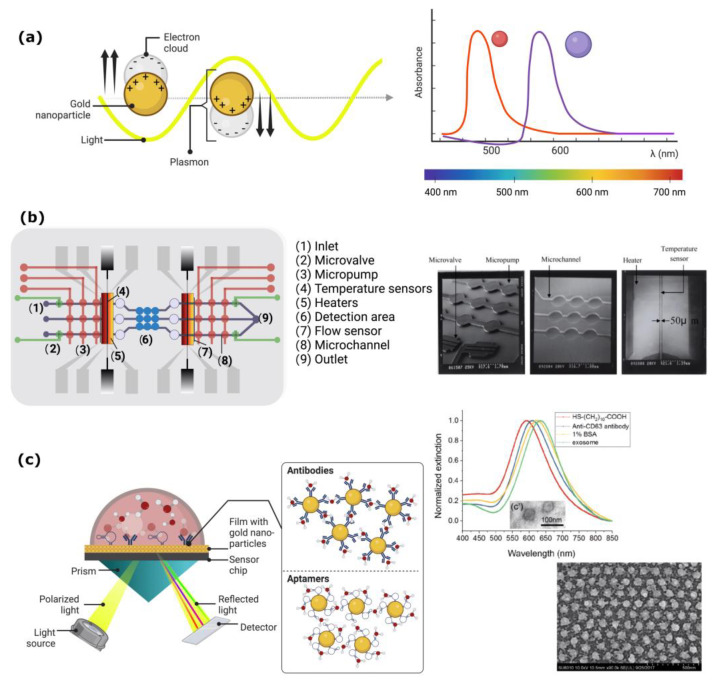
(**a**) Schematic configuration of a surface plasmon resonance biosensor. A visual representation of the electron cloud’s behavior in a thin gold film when stimulated by light, resulting in the surface plasmon effect, indicated by an absorption peak at a specific frequency that varies based on the size and shape of the nanoparticles. (**b**) This micrograph illustrates the different zones of Lee’s microfluidic channels (reproduced with permission) used for the operation of a lab-on-a-chip for immunoassays and detection of plasmonic signals [74]. (**c**) The presented scheme depicts the process occurring in the SPR sensor. Resonance produces an amplified increase in the extinction signal when gold nanoparticles bind to antibodies, enabling the detection of the immune signal that identifies the presence of cancerous cells in the sample. Adapted with permission from Ref. [75]. Copyright 2019 American Chemical Society. (Created with Biorender.com).

The interaction between light and metallic surfaces (SPR) or metallic nanoparticles (LSPR) leads to strong confinement of electromagnetic field intensity, resulting in increased sensitivity. SPR results from the resonant oscillation of conduction electrons at the interface between materials with negative and positive permittivity, which is stimulated by incident light. An example of an SPR biosensor is shown in Figure 4, where the incident light is totally internally reflected from the prism and creates evanescent waves outside the prism that interact with the plasma waves on the metal surface (usually Au or Ag) and trigger the plasmon resonance. The angle of minimum reflection, which is very sensitive to the refractive index of biomaterials adsorbed onto the metal surface, can be used to observe SPR and detect molecular adsorption, such as polymers, DNA, proteins, or molecular interactions [73].

Figure 4 illustrates a sensor chip consisting of a thin gold layer that is illuminated from behind by polarized light from a laser through a prism. The light reflects off the metal film, acting as a mirror, and the intensity of the reflected light can be used to monitor changes in the angle of incidence. At a specific angle of incidence, the intensity of the reflected light reaches a minimum, and the light excites the surface plasmons, resulting in a drop in the reflected light’s intensity. The location of this minimum angle can be determined by the properties of the solution interface. This sensitive technique enables the monitoring of adsorption phenomena and reaction kinetics without the need for molecule labeling. The benefits of this method include its high sensitivity, temporal resolution, and the elimination of time and cost associated with labeling, as well as potential disturbances during biorecognition studies. However, the main drawbacks of this method are its complexity, requiring specialized personnel and a high cost, and the large size of currently available instruments.

The potential of SPR to characterize thin films and monitor processes at metal interfaces was identified in the late 1970s. In 1995, Nylander and Liedberg [76] demonstrated the use of SPR for gas detection and biosensing by using a system with an organic layer that absorbed the anesthetic gas halothane. They showed that the method was sensitive to changes in the film’s optical properties upon gas exposure down to the parts per million (ppm) range. They also noted that SPR detection was just one possibility and that many other new optical methods for real-time biospecific interaction analysis would be developed. Since then, SPR sensing has received growing attention from the scientific community and has been identified as having an excellent potential for affinity biosensors. It allows real-time analysis of biospecific interactions without the need for labeled molecules. Several companies have commercialized SPR sensor technology, making it a leading technology for direct real-time observation of biomolecular interactions.

SPR biosensors are a popular option for analyzing biomolecular interactions in biomedical research due to their label-free, real-time, and high-throughput capabilities. This technique utilizes the resonance conditions of surface plasmons, which are influenced by the dielectric properties of biomolecules at the interface [65,77]. The thin gold film-based SPR sensor is widely used for drug discovery, antibody characterization, proteomics, immunogenicity, and biotherapeutic development by both academia and pharmaceutical companies. Additionally, more versatile and user-friendly SPR biosensors, such as those using gold nanoparticles, are being developed for various applications, including POC diagnostics [78]. With the ability to observe binding events in real-time, determine analyte concentrations, and calculate binding constants, the SPR biosensor shows promise as a valuable tool in biomedical research [72].

Functionalized gold nanorods in an aqueous solution have been used as an example of multiplexed detection, where assays can detect changes in the intensity and displacement of the longitudinal surface plasmon band. These changes result from a dependent response to binding events between requirements and antigens. While these studies have demonstrated the detection of multiple assays within a single assay, further improvement is needed in terms of reproducibility, detection limit, and spatial resolution. This could be achieved by using detectors with better noise ratios or higher resolution, or by increasing the intrinsic sensitivity of the biosensor [79]. An alternative approach to enhancing sensitivity involves using different shapes of nanoparticles or detecting individual NPs [80], which can result in narrower plasmon bands. However, the lower signal-to-noise ratio could limit the efficacy of this approach.

Integrating SPR sensing technology with microfluidics offers several benefits, such as automation, reduced sample volumes, quick processing, and potentially improved sensing efficiency through proper design [74]. Microfluidics has the potential to speed up reaction rates, reduce diffusion times, and improve surface regeneration. The use of microfluidics automation leads to increased reproducibility and improved precision in controlling reaction stages. The integration of sensing and microfluidic systems is critical for achieving high performance, particularly in parallel SPR imaging, where the sample interacts simultaneously with multiple ligands immobilized on a single chip. Microfluidics can increase the throughput of a single chip and ensure better sample flow delivery. Various microfluidic formats utilized for surface plasmon resonance biosensors in existing literature are summarized in Table 2.

Table 3 summarizes the most widely used SPR techniques for cancer cell detection. Future LSPR biosensors will likely feature novel nanomaterials, receptors, and sensing devices. The trend in research is shifting toward multimodal biosensors, which utilize multiple sensing methods for biomolecule detection instead of single-mode sensing devices. These sensing methods have also been combined with microfluidic and CMOS devices to provide real-time, continuous monitoring of biomolecules. Additionally, recent advancements in synthetic receptor design and screening through selection methods and click chemistry are expected to result in more sensitive and specific detection of various biomarkers. These breakthroughs will lead to the development of new technologies and materials for disease diagnosis and environmental monitoring. A recent study used LRSPP biosensors to detect biomarkers associated with B-cell tumors in patients. Despite some quantitative differences in laboratory data, the results showed a clear distinction between normal and leukemic sera. This study highlights the potential of LRSPP biosensors as a cost-effective and rapid solution for B-cell tumor screening in clinical diagnostics, especially with the possibility of miniaturization.

## 6. Surface-Enhanced Raman Scattering

Raman scattering is an important inelastic scattering process utilized for the detection and characterization of biomaterials. Although it provides molecular-specific information, it suffers from low sensitivity. Surface-enhanced Raman scattering (SERS) is a technique that enhances the Raman signal and enables highly sensitive detection of molecules adsorbed on a specific medium or interface [86]. Here we provide an overview of SERS, including the underlying mechanisms of signal enhancement and the recent developments in SERS-based biosensing. SERS biosensing is a powerful tool that enables sensitive and selective detection of a broad range of analytes, including nucleic acids, proteins, and small molecules, with excellent molecular specificity [87]. SERS is a surface-sensitive technique that enhances Raman scattering signals by several orders of magnitude, leading to significantly improved sensitivity. The enhancement of the Raman signal is achieved through the interaction of the incident photons with localized surface plasmons (LSPs) and chemical processes at the interface between the metal and the analyte [88].

SERS signal enhancement arises from two mechanisms, namely electromagnetic and chemical. Electromagnetic enhancement occurs through the excitation of LSPs in metallic nanoparticles or surfaces, resulting in a localized electric field enhancement [86]. The LSPs, which are collective oscillations of free electrons in the metal nanoparticles, generate an electromagnetic field that is confined in the vicinity of the surface. The electric field intensity at the metal surface decays exponentially with distance, making SERS a highly surface-sensitive technique [89]. Chemical enhancement, on the other hand, arises from charge transfer and resonance Raman scattering between the analyte and the metal surface. This enhancement mechanism depends on the nature of the analyte and the metal surface, making it a more complex mechanism to understand and control [90]. Several theories have been proposed to explain the chemical enhancement, including the formation of a charge–transfer complex between the analyte and the metal surface and the modification of the analyte’s molecular orbitals [91].

**Figure 5:**
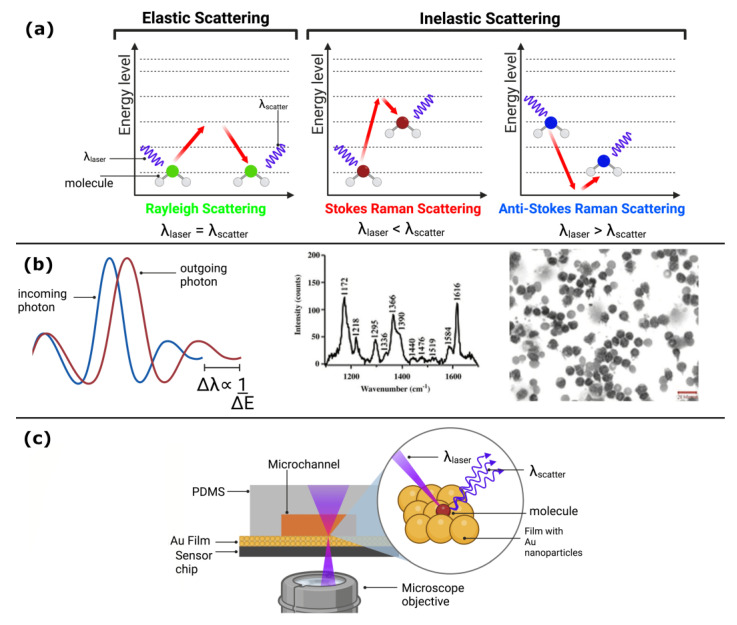
(**a**) Energetic levels in SERS: understanding the interaction between molecules and plasmonic nanoparticles. (**b**) Bright field accompanying with Raman spectra of chronic lymphocytic leukemia cells stained with Giemsa and labeled with anti-CD19-SERS nanoparticles. Reproduced with the permission of [99]. (**c**) Integrated microfluidic and biosensing SERS system for high sensitivity detection in biomedical applications. (Created with Biorender.com).

Over the past decade, SERS-based biosensing has emerged as a highly promising tool for the detection of a wide range of biomolecules with high sensitivity and selectivity. SERS has been integrated with various platforms, including microfluidics, lab-on-a-chip devices, and paper-based sensors, enabling the rapid and cost-effective detection of analytes [92]. For example, SERS-based biosensors have been developed for the detection of cancer biomarkers, viruses, bacteria, and toxins [93]. Moreover, the use of SERS in vivo imaging and clinical diagnostics has been explored, offering great potential for future biomedical applications. Figure 5 implements a SERS microfluidic system for cancer recognition.

The use of metal nanoparticles In SERS-based CTC detection has become a popular method due to their ability to enrich and isolate CTCs in the bloodstream. However, accurately locating the focal point of the laser on SERS-tagged cells remains challenging due to the mobility of CTCs [94]. To address this issue, magnetic nanoparticles have been introduced as a crucial element in the design of SERS probes. Xiong et al. demonstrated the use of magnetic nanochains to magnetically control cell binding with SERS probes within a microfluidic channel [95]. Another alternative approach for detecting circulating cancer stem cells (CCSCs) using SERS has been proposed by Cho et al. They designed a unique set of SERS probes with specific sets of antibodies, Raman dye, and a double-stranded DNA linker. One of the DNA linkers was conjugated with an anti-CD133 antibody, allowing for CCSCs isolation [96]. Similarly, Willner et al. developed SERS droplet microfluidics for the single-cell analysis of CTCs [97], while Pallaoro et al. integrated a microfluidic system using SERS to identify and quantify cancer cells from a mixed population of cells flowing through a microfluidic channel. Despite these developments, the elimination of unwanted SPR signals and artifacts is still necessary in the detection of CTCs using SERS [98]. These investigations have effectively showcased the potential of SERS in improving the detection of cancer biomarkers, quantifying cancer cells, and isolating CCSCs from a heterogeneous mixture of cells through the utilization of SERS-based probes and microfluidic devices.

The integration of Raman spectroscopy with optical cell manipulation, or “Raman tweezers,” has been extensively used for several applications. These include distinguishing between biotic and abiotic particles, identifying protein expression in living bacteria, sorting cells based on Raman identification, and detecting hyperosmotic stress in single trapped yeast cells. Compared to optical stretcher techniques, Raman tweezers have shown greater specificity in discriminating normal blood cells from CTCs [100]. Higher spectral intensities at specific wavelengths representing intracellular proteins and nucleic acids have been observed in cancerous cells, including colorectal epithelial cells, prostate cancer cells, cancerous hematopoietic cells, and astrocytoma, as compared to normal cells [101].

Optical trapping waveguides combined with microfluidic channels offer an ideal platform for Raman tweezers. By controlling the flow of reagents, optofluidic systems provide an efficient way of aligning the waveguides with the channels. For example, Zachariah et al. utilized an integrated optofluidic system for Raman tweezers to trap and sort individual cells based on their Raman spectra [102]. The researchers were able to detect the resonance Raman spectra of a single trapped cell at different excitation wavelengths, enabling Raman signal-activated sorting of leukocytes and three other tumor cell lines.

The combination of Raman tweezers and microfluidics can facilitate the real-time observation of cellular responses to environmental stimuli or drug delivery. For instance, Ramser et al. employed Raman tweezers in a microfluidic channel to monitor the oxygenation dynamics of a single red blood cell [103]. They detected time-variations of Raman spectral peaks, which resulted from the specific vibrational resonance of the porphyrin groups of the RBC hemoglobin. With this optofluidic platform, it is possible to perform long-term, in vivo, and dynamic characterization of the oxygenation cycle of a single RBC.

## 7. Discussion

Optical biosensing techniques, such as fluorescence, chemiluminescence, SPR, and SERS are widely used in lab-on-a-chip devices due to their high sensitivity and specificity. Fluorescence-based biosensors use fluorescent probes to detect the presence of specific biomolecules, and the fluorescence signal is read by an integrated detector. Chemiluminescence-based biosensors produce light through a chemical reaction, which is then detected by the integrated detector. SPR biosensors measure changes in the refractive index of a sample near a metal surface. SERS biosensors use surface-enhanced Raman scattering to detect the presence of specific biomolecules. All of these techniques are particularly useful for detecting low concentrations of analytes.

There are other optical-based techniques for biosensing, like multi-photon, photochemical and impedance biosensors. Multi-photon biosensors use nonlinear optical processes to excite fluorescent molecules, which can be used to detect specific biomolecules [104]. Photochemical biosensors use light to trigger a chemical reaction, which can be detected by an integrated detector [105]. Impedance biosensors measure changes in the electrical properties of a sample, which can be used to detect specific biomolecules [106,107]. These techniques offer some benefits, such as high spatial resolution and low cost, but they have typically lower sensitivity compared to the optical biosensors presented here.

While optical biosensing techniques, such as fluorescence, chemiluminescence, surface plasmon resonance, and SERS have been successfully integrated into lab-on-a-chip devices, multi-photon, photochemical, and impedance biosensors have not been widely adopted for such applications. One of the main reasons for this is the complexity of the instrumentation required to perform these types of measurements. Multi-photon and photochemical biosensors require sophisticated laser systems and optics, which can be challenging to miniaturize and integrate into a lab-on-a-chip device. Impedance biosensors require specialized electronics for signal processing, which can also be challenging to integrate into a small, portable device. Another reason is that these techniques often require complex sample preparation or labeling, which can be difficult to perform on a microfluidic scale. Despite these challenges, researchers are continuing to explore ways to overcome these limitations and develop lab-on-a-chip devices that incorporate these powerful biosensing techniques [108].

## 8. Future Perspectives

The development of biosensing methods using lab-on-a-chip devices has opened new possibilities for faster and more sensitive detection of biological analytes. Among the optical techniques used in these devices, fluorescence, chemiluminescence, surface plasmon resonance, and SERS have shown great potential for various applications, such as POC in cancer diagnostic and treatment.

SPR is an effective and cost-efficient label-free biosensing method. In addition to the traditional planar metal film on a glass substrate, alternative formats, such as optical fiber and grating-based excitation methods, have been developed. Metal nanostructures have also been utilized for sensing, making it possible to reduce the sensor size for POC applications. The use of microfluidics further enhances SPR sensing efficiency, and it can even be performed on paper. However, to eliminate unwanted SPR signals and artifacts, sample pre-processing is required. The integration of advanced microfluidic components for separation and extraction may ultimately allow SPR sensors to function as a complete POC diagnostic device in the future.

Looking at the future, there are several promising directions for research and development in the field of lab-on-a-chip biosensors. One major area of focus is the integration of multiple biosensing techniques into a single device. By combining multiple detection methods, it may be possible to improve sensitivity, specificity, and multiplexing capabilities of the biosensor. For example, combining fluorescence with SERS could allow for simultaneous detection of multiple biomarkers with high sensitivity and specificity [109].

Another area of future work is the development of biosensors with improved portability and ease of use. This includes not only miniaturization of the device itself but also simplification of the sample preparation and detection processes. For example, researchers are exploring ways to perform sample preparation steps, such as DNA amplification or protein separation, diagnostics for malaria elimination, and Coulter counter using a modular platform directly on the lab-on-a-chip device, eliminating the need for external equipment and reducing the time required for analysis [110,111].

In addition, the integration of biosensors with microfluidics and nanotechnology is another promising area of research. Microfluidics can provide precise control over sample flow and mixing, while nanotechnology can enhance the sensitivity of the biosensor by increasing the surface area available for binding with the analyte. For example, the integration of plasmonic nanoparticles with SERS biosensors has been shown to improve sensitivity by several orders of magnitude [112,113,114].

Finally, the development of biosensors with the ability to detect a wide range of analytes, including small molecules, proteins, and cells, is a critical area for future work. While many biosensors are currently limited to the detection of a specific target, the development of more versatile biosensors could greatly expand their utility in a variety of fields. The field of lab-on-a-chip biosensors is rapidly advancing, with numerous promising directions for future research and development. By continuing to improve the sensitivity, specificity, portability, and versatility of these devices, it is likely that they will become increasingly important tools for applications in healthcare and cancer.

## 9. Conclusions

Lab-on-a-chip devices have shown significant promise in the detection, isolation, and processing of CTCs. However, several challenges must be addressed for their use in clinical trials, patient diagnosis, and prognosis. Sensitivity and reliability are critical in CTC detection, and false-positive and false-negative results must be minimized. Researchers need to investigate the effect of dead CTCs on the CTC count statistics and explore more efficient markers, such as aptamers. Nanotechnology can potentially address the heterogeneity of CTCs and develop patient-specific CTC diagnostics. Standard operating procedures are necessary to ensure reproducibility and reliability of CTC detection and enumeration using lab-on-a-chip devices across different testing labs. The integration of lab-on-a-chip devices can also improve sample handling, reduce pre-analytical variation, and enhance detection as well as reproducibility and reliability for enumeration.

Chemiluminescence-based biosensors are affordable, simple, and have a low limit of detection. Electrogenerated chemiluminescence sensors have shown exceptional sensitivity, wide dynamic range, and excellent controllability. Nanomaterials have expanded the range of applications and improved sensitivity. Fluorescence and chemiluminescence have been applied as detection methods for sandwich EIAs, such as ODI CLEIA, to enhance sensitivity. Chemiluminescence has also shown promise for bioimaging and therapy due to its excellent characteristics, although several issues still need to be addressed. Future research should focus on the development of chemiluminescence therapy systems activated by various tumor- or disease-associated biomarkers and the exploration of the use of chemiexcited photothermal therapy platforms for oncotherapy.

Significant developments in SPR technology highlight its key application in medical areas, and provide examples of its use in biosensing. Despite their promising features, fabricating these biosensors presents significant challenges, including minimizing non-specific binding, cross-reactivity of the target set, noise reduction, synthesizing stable nanoparticles, and selecting well-characterized bioreceptors. SPR biosensors have become a popular choice for label-free, real-time, and high-throughput analysis of biomolecular interactions in biomedical research.

Finally, automated diagnostic platforms for POC testing, such as microfluidics technology, have the potential to greatly improve early detection and treatment of diseases in developing countries. However, more research is necessary to ensure their reliability, sensitivity, and affordability. The application of lab-on-a-chip devices and chemiluminescence-based biosensors is still in its infancy and holds great promise for improving disease detection, monitoring, and treatment. With continued research and development, these technologies have the potential to revolutionize healthcare and improve patient outcomes.

## Figures and Tables

**Table 1:** Overview of some lab-on-a-chip chemiluminescence biosensors for cancer detection: design, dynamic range, and detection limit of different biosensing strategies.

Luminophore Type	Sensor Model	Analyte	Sensing System	Dynamic Range	Detection Limit	Ref.
Graphene quantum dots	Sandwich-type immunosensor	carcinoembryonic antigen	signal amplification strategy based on P5FIn/erGO	0.1 pgmL^−1^–10 ng mL^−1^	3.78 fg mL^−1^	[55]
Luminol	ECL sensor	human breast cancer cells (MCF-7)	bipolar electrode mounted into 3D printed microchannel	100–700 cells	10 cells	[56]
			paper-based closed bipolar electrode	1.0 × 10^2^–1.0 × 10^7^ cells mL^−1^	40 cells mL^−1^	[57]

Table 1 is reprinted with permission from Ref. [58], copyright 2019 Elsevier.

**Table 2:** Summary of various microfluidic SPR sensors formats.

SPR Sensor Type	Microfluidic Formats	Detection Limits	Required Volume	Analysis Time
Prism-based SPR sensor	Flow-through cell	0.2 µg/mL	100–1000 µL	5–20 min
	Digital microfluidic	1 µg/mL	0.2–1 µL	1 min
Waveguide, fiber-optic SPR sensor	Flow-through cell	100 µg/mL	~200 µL	10 min
Grating-based SPR sensor	Flow-through cell	100 µg/mL	-	10 min
	CD-based	200 µg/mL	20–40 µL	5 min
Localized SPR sensor using nanostructures	Capillary-driven (paper and membrane-based)	-	-	-
	Flow-through cell	0.3–1 µg/mL	30–200 µL	10 min

Table 2 is reproduced with permission from Ref. [81]. Copyright 2016 by the authors; licensee MDPI, Basel, Switzerland.

**Table 3:** Examples of nanostructure-based LSPR biosensors for the detection of various molecules ^1^.

Classification	Substrate	Receptor	Analyte	Linear Range, LOD	Assay Time	Real Sample	Features	Ref.
NP-coated optic fiber-based platform	Au film-coated optical fiber	Aptamer, HER2 antibody	Breast cancer HER2 protein	9.3 ng/mL (77.4 pM)	10 min	ND	HER2 biomarker detection using sandwich assay with anti-HER2 ssDNA aptamer and HER2 antibody.	[82]
	Optical fiber with copper oxide nanoflower (CuO-NF) and Au NPs-coated Gox structure	2-deoxy-D-glucose (2-DG)	Cancer cell	1 × 10^−2^–1 × 10^6^ cells/mL, 2–10 cells/mL	ND	ND	Use of multi-core fiber structure. Coating of optical fiber with GOx and CuO-NF: increasing surface area and adsorption capability. Discrimination of cancer cells using 2-DG that binds to GULP receptor: the presence of more GULP receptors on cancer cell, inducing a peak shift. Reusable through washing with PBS.	[83]
Solid-based nanopatterned flatform	Au nanopillars on quartz coverslips	Anti-CD63 antibody	Exosome	ND	ND	MCF7 breast adenocarcinoma cells	Fabrication of Au nanopillar array by electron beam lithography. Enabled multiplexed measurement using LSPRi.	[84]
	Au nano-ellipsoid array on quartz substrate	Anti-CD63 antibody	Exosome	1 ng/mL	<4 h	ND	Fabrication of nanostructures via AAO-templated Au deposition on a quartz substrate. Integration of LSPR and microfluidic systems.	[75]
	Metal-insulator metal (MIM) nanodisks on PDMS	none	Cancer cell (adherent cell)	NA	ND	ND	Construction of a MIM nanodisk consisting of Au-SiO_2_-Au on an InP substrate. Fabrication of a flexible sensor by transferring a MIM nanodisk onto PDMS.	[85]

^1^ Abbreviations: AuNP, gold nanoparticle; LSPRi, LSPR imaging; NP, nanoparticle; ND, Not determined.

## Data Availability

No new data were created in this study. Data sharing is not applicable to this article.
